# Association Between Hearing and Vision Impairment and Risk of Dementia: Results of a Case-Control Study Based on Secondary Data

**DOI:** 10.3389/fnagi.2019.00363

**Published:** 2019-12-20

**Authors:** Bernhard Michalowsky, Wolfgang Hoffmann, Karel Kostev

**Affiliations:** ^1^German Center for Neurodegenerative Diseases (DZNE) Site Rostock/Greifswald, Greifswald, Germany; ^2^Institute for Community Medicine, Section Epidemiology of Health Care and Community Health, University Medicine Greifswald (UMG), Greifswald, Germany; ^3^IQVIA, Epidemiology, Frankfurt, Germany

**Keywords:** dementia, hearing impairment, sensory impairments, vision loss, case-control study

## Abstract

**Introduction**: Hearing and vision loss are highly prevalent in elderly adults, and thus frequently occur in conjunction with cognitive impairments. Studies have shown that hearing impairment is associated with a higher risk of dementia. However, evidence concerning the association between vision loss and dementia, as well as the co-occurrence of vision and hearing loss and dementia, has been inconclusive.

**Objectives**: To assess the association between: (i) either hearing or vision loss and the risk of dementia, as well as between; and (ii) the combination of both sensory impairments and the risk of dementia.

**Methods**: This case-control study was based on a 5-year data set that included patients aged 65 years and older who had initially been diagnosed with dementia diseases by one of 1,203 general practitioners in Germany between January 2013 and December 2017. In total, 61,354 identified dementia cases were matched to non-dementia controls, resulting in a sample size of 122,708 individuals. Hearing loss and vision loss were identified using the ICD-10 diagnoses documented in the general practitioners’ files prior to the initial dementia diagnosis. Multivariate logistic regression models were fitted to evaluate the associations between visual and/or hearing impairment and the risk of dementia and controlled for sociodemographic and clinical variables.

**Results**: Hearing impairment was documented in 11.2% of patients with a dementia diagnosis and 9.5% of patients without such a diagnosis. Some form of vision impairment was documented in 28.4% of patients diagnosed with dementia and 28.8% of controls. Visual impairment was not significantly associated with dementia (OR = 0.97, CI = 95% 0.97–1.02, *p* = 0.219). However, patients with hearing impairment were at a significantly higher risk of developing dementia (OR = 1.26, CI = 95% 1.15–1.38, *p* < 0.001), a finding that very likely led to the observed significant association of the combination of both visual and hearing impairments and the risk of dementia (OR = 1.14, CI = 95% 1.04–1.24, *p* = 0.005).

**Discussion**: This analysis adds important evidence that contributes to the limited body of knowledge about the association between hearing and/or vision loss and dementia. It further demonstrates that, of the two, only hearing impairment affects patients’ cognition and thus contributes to dementia risk.

## Background

Aging populations are causing a rapid increase in the number of people affected by age-associated illnesses, such as dementia diseases (Prince et al., 2014; Michalowsky et al., 2018). Worldwide, more than 47 million people are currently living with dementia (PwD), and that number will increase to 75 million by 2030 (Prince et al., 2013). The worldwide annual cost of dementia is estimated to be $818 billion (Wimo et al., 2017). Therefore, dementia diseases currently represent, on average, one-third of the total societal cost of elderly care, demonstrating that dementia diseases constitute a healthcare priority (Michalowsky et al., 2019).

In the absence of a cure, patients need a timely diagnosis and evidence-based treatment and care in order to control complications, delay the progression of dementia, and improve patient-related outcomes [Deutsche Gesellschaft für Allgemeinmedizin und Familienmedizin e.V.(DEGAM) (2008); Deutsche Gesellschaft für Psychiatrie, Psychotherapie und Nervenheilkunde (DGPPN) (2015)]. Furthermore, in addition to target the delay of disease progression, preventing or delaying the onset of dementia is key to tackle the existing healthcare crisis. Several studies have provided substantial evidence of modifiable risk factors (Kivipelto and Solomon, 2009; Fratiglioni and Qiu, 2011; Mangialasche et al., 2012), including active engagement in social and physical activities, as well as mentally stimulating activities, all of which can determine the risk of late-life dementia. More recently, hearing and vision loss are evaluated as possible modifiable risk factors of dementia diseases. Both impairments are very common in the elderly, and their prevalence increases with age (Wilson et al., 1999; Agrawal et al., 2008; Bourne et al., 2013; Flaxman et al., 2017; Homans et al., 2017). Hearing loss is present in two-thirds of adults aged 70 and older (Lin et al., 2011a,b,c) and thus very frequently occurs in conjunction with cognitive impairment. The same applies to vision loss, for which the leading cause is an uncorrected refractive error or various other conditions, such as cataracts, age-related macular degeneration, glaucoma, and diabetic retinopathy (Gohdes et al., 2005; Albers et al., 2015).

Previous evidence has shown that hearing impairment is associated with a decrease in cognition and a higher risk of dementia diseases. The meta-analysis of Wei et al. (2017) aggregated data on more than 15,000 subjects from different cohort studies, revealing that hearing loss was associated with a greater risk for cognitive impairment (RR 1.3, CI 95% 1.12–1.51) and dementia (RR 2.4, CI 95% 1.58–3.61). This was confirmed in a second meta-analysis recently published by Loughrey et al. (2018) aggregating different cohort and cross-sectional studies.

In addition, an uncorrected refractive error, for example, could also lead to neuropathological changes (Albers et al., 2015). Therefore, both sensory impairments could be associated with decreased cognitive function. Based on a retrospective cohort study, Davies-Kershaw et al. (2018) revealed that moderate and severe vision loss was significantly associated with an increased risk of dementia. However, despite this and other evidence (Reyes-Ortiz et al., 2005; Clemons et al., 2006; Pham et al., 2006; Rogers and Langa, 2010; Davies-Kershaw et al., 2018), longitudinal studies have shown inconclusive evidence concerning the association between vision loss and the risk of dementia (Lin et al., 2004; Reyes-Ortiz et al., 2005; Sloan et al., 2005; Valentijn et al., 2005; Fischer et al., 2016; Hong et al., 2016; Naël et al., 2019). Most studies have focused on the cognitive decline rather than on dementia, used varied diagnostic criteria, or have been based on a subjective rather than an objective assessment, using small sample sizes or cross-sectional data.

According to the results of recent studies, the combination of both of these age-related sensory impairments, i.e., vision and hearing loss, could result in a higher risk of cognitive decline and dementia diseases. Based on a prospective cohort study of more than 6,000 women aged 69 and older, Lin et al. (2004) revealed that vision loss—but not a hearing loss—was associated with cognitive and functional decline, which is not in line with the existing evidence for the effect of hearing impairment. A combination of both sensory impairments was found to be significantly associated with the greatest odds for cognitive decline in this study. Contrary to this finding, Hong et al. (2016) demonstrated, based on a population study of more than 3,500 participants, that dual sensory impairment was not significantly associated with a subsequent decline in cognition.

Therefore, the evidence is currently inconclusive, and there is a scarcity of knowledge regarding the additive combination of both sensory impairments and their association with a higher risk of dementia. This study aimed to assess: (i) the association between either hearing or vision loss and the risk of dementia separately, as well as; and (ii) the association of the additive combination of both sensory impairments—hearing and vision loss—and the risk of dementia, using a large representative sample of more than 120,000 German adults aged 65 and older and an objective measure of sensory impairment.

## Materials and Methods

### Overview

This case-control study was based on a 5-year data set that included patients aged 65 years and older who had initially been diagnosed with dementia by one of 1,203 general practitioners in Germany (cases) between January 2013 and December 2017 (index date) and patients without any dementia diagnoses during this time period (controls). In total, *n* = 61,354 identified dementia cases were matched to non-dementia controls, resulting in a sample of 122,708 patients with or without dementia. Hearing loss and vision loss (diagnosed prior to the dementia diagnosis) were identified using the ICD-10 diagnoses documented in the general practitioners’ files. Logistic regression models were conducted to evaluate the association between visual and/or hearing impairment and dementia.

### Database

This study was based on data from the Disease Analyzer database (IQVIA), which compiles drug prescriptions, diagnoses, and basic medical and demographic data obtained directly and in anonymous format from computer systems used in the practices of general practitioners and specialists (Rathmann et al., 2018), covering about 3% of all outpatient practices in Germany. Diagnoses [International Classification of Diseases, 10th revision (ICD-10)], prescriptions [Anatomical Therapeutic Chemical (ATC) Classification system], and the quality of reported data are monitored by IQVIA based on a number of criteria (e.g., completeness of documentation, the linkage between diagnoses and prescriptions). In Germany, the sampling methods used to select the practices are appropriate for obtaining a representative database of primary and specialized care practices (Rathmann et al., 2018).

### Study Population, Sample Size, Participant Flow, and Drop Out

**Figure 1:**
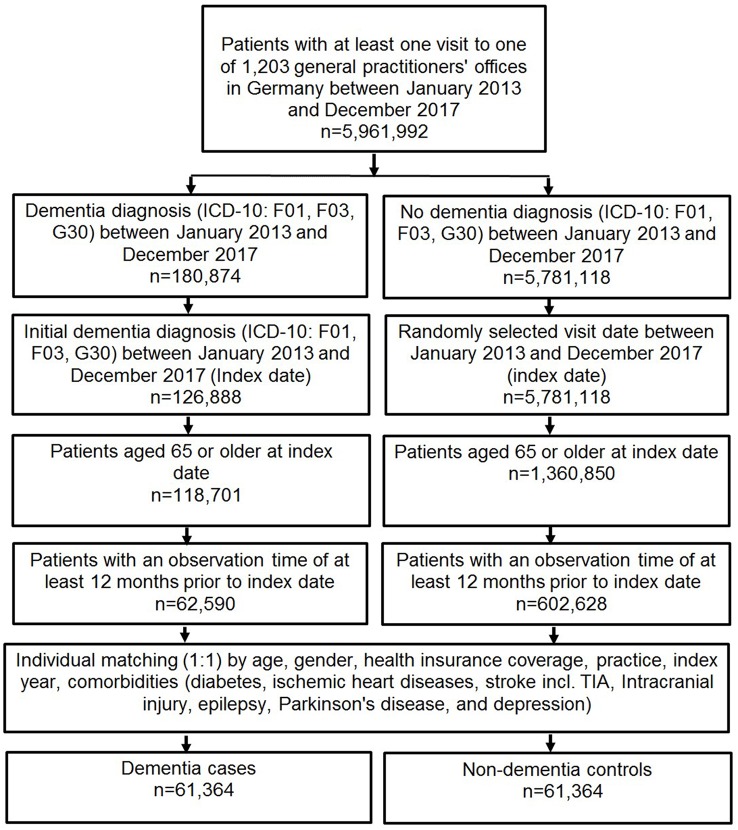
Study flow chart.

This case control-study included patients diagnosed with dementia (ICD-10: F01, F03, G30, F06.7) by one of 1,203 general practitioners in Germany between January 2013 and December 2017 (Figure 1). Inclusion criteria were as follows: age ≥65 years at the index date; observation time of at least 12 months prior to the index date.

After applying similar inclusion criteria, dementia cases were matched to non-dementia controls by age, sex, health insurance coverage, practice, index year (year of dementia diagnosis), and comorbidities (diabetes, ischemic heart diseases, stroke (including transient ischemic attack), intracranial injury, epilepsy, Parkinson’s disease, and depression). For the controls, the index date was that of a randomly selected visit between January 2013 and December 2017 (Figure 1). Overall, the present study included a total of 122,708 patients, comprising 61,354 patients with dementia and 61,354 patients without dementia.

### Study Outcomes and Variables

The main outcomes of the study were the associations between objective hearing and visual impairment and dementia. As described above, the following ICD-10 diagnoses received on the index date were used to identify patients with dementia: F01, F03, G30, and F06.7. In order to identify patients with hearing impairment prior to the initial dementia diagnosis, as well as categorize hearing impairment, one of the following two diagnoses documented by general practitioners was used: conductive and sensorineural hearing loss (ICD-10 diagnosis: H90) or other hearing loss (H91). To identify patients with visual impairment prior to the dementia diagnosis, one or more of the following diagnoses were used: disorders of lens (H25–H28), disorders of choroid and retina (H30–H36), glaucoma (H40–H42), disorders of vitreous body and globe (H43–H45), disorders of optic nerve and visual pathways (H46–H48), disorders of ocular muscles, binocular movement, accommodation and refraction (H49–H52), visual disturbances (H53), and visual impairment including blindness (H54).

Furthermore, socio-demographic data, including age, sex, health insurance coverage, and co-existing morbidities documented prior to the dementia diagnosis, such as diabetes mellitus (E10–14), ischemic heart disease (I20–I25), stroke including TIA (I60–I64, G45), intracranial injury (S06), epilepsy (G40, G41), Parkinson’s disease (G20, G21), and depression (F32, F33) were assessed.

### Statistical Analyses

To validate the matching procedure, differences in the sample characteristics between those with and those without a dementia diagnosis were tested by using chi-squared tests for categorical variables and Wilcoxon tests for continuous variables. Multivariate logistic regression models were used to evaluate the association between visual and hearing impairment and dementia incidence. The association between any or a specific visual or hearing impairment diagnosis and the risk of dementia were evaluated separately. That means that, for each hearing or vision impairment diagnosis, a separate model was used to test the association between each specific or any impairment and dementia incidence. In addition, to evaluate the association between hearing and vision loss and dementia incidence compared to healthy controls without vision or hearing impairment, patients were classified into one of the following four groups: only hearing impairment, only visual impairment, both hearing, and visual impairment, and no hearing and no visual impairment (reference group). All models were adjusted for age, sex, health insurance coverage, and the following comorbidities: diabetes mellitus, ischemic heart disease, stroke, intracranial injury, epilepsy, Parkinson’s disease, and depression. Analyses were carried out using SAS version 9.4.

### Ethical Aspects

German law allows for the use of anonymous electronic medical records for research purposes under certain conditions. According to German legislation, it is not necessary to obtain informed consent from patients or approval from a medical ethics committee for this type of observational study, which contains no directly identifiable data. Therefore, no protected health information was available for queries and no Institutional Review Board (IRB) approval was required for the use of this database or the completion of this study.

## Results

Patients were on average 81 years old, the majority were female (61%), and more than one-third were suffering from diabetes (41%), ischemic heart disease (39%), or depression (32%). The matching procedure successfully resulted in non-significant differences in socio-demographic and clinical variables between patients with dementia diseases and non-dementia controls. A description of the sample is given in Table 1.

**Table 1:** Basic characteristics of study patients (after 1:1 matching by age, sex, health insurance coverage, physician, index year, and comorbidities).

	Proportion among patients with dementia (%; *n* = 61,354)	Proportion among patients without dementia (%; *n* = 61,354)	*p*-value
Age
Mean, SD	80.7 (6.8)	80.7 (6.8)	1.000
Age 65–69	5.5	5.5	1.000
Age 70–79	38.3	38.3	
Age 80–89	45.2	45.2	
Age ≥90	11.0	11.0	
Sex		
Female	61.0	61.0	1.000
Male	39.0	39.0	
Insurance		
Private health insurance coverage	4.9	4.9	1.000
Statutory health insurance coverage	95.1	95.1
Comorbidities *(documented diagnoses within 12 months prior to the index date)*			
Diabetes mellitus (E10–14)	40.6	40.6	1.000
Ischemic heart disease (I20–I25)	38.5	38.5	1.000
Stroke incl. TIA (I60–I64, G45)	15.6	15.6	1.000
Intracranial injury (S06)	1.5	1.5	1.000
Epilepsy (G40, G41)	2.6	2.6	1.000
Parkinson’s disease (G20, G21)	4.4	4.4	1.000
Depression (F32, F33)	32.2	32.2	1.000

### Prevalence of Hearing and Vision Loss

Table 2 represents the proportions of patients with and without dementia and with documented visual and hearing impairment diagnoses prior to the dementia diagnosis. Hearing impairment was documented in 11.2% of patients with and 9.5% of patients without dementia. Another hearing loss (ICD-10 H91) was documented most frequently (10.4% vs. 8.9%), while the conductive and sensorineural hearing loss was reported less frequently (1.2% vs. 0.9%) in patients with dementia than in patients without dementia. However, both were found more often in these patients than in non-dementia controls.

**Table 2:** Prevalence of hearing and vision loss and their association with dementia in general practices in Germany.

Diagnosis (ICD-10 Code)	Proportion among patients with dementia (%)	Proportion among patients without dementia (%)	Odds ratio (95% CI)	*p*-value
Hearing impairment
Any hearing impairment (H90, H91)	11.2	9.5	1.21 (1.13–1.29)	<0.001
Conductive and sensorineural hearing loss (H90)	1.2	0.9	1.27 (1.05–1.54)	0.015
Other hearing loss (H91)	10.4	8.9	1.20 (1.12–1.28)	<0.001
Visual impairment				
Any visual impairment (H25–H54)	28.4	28.8	0.98 (0.94–1.03)	0.434
Disorders of lens (H25–H28)	12.6	12.7	1.00 (0.94–1.06)	0.905
Disorders of choroid and retina (H30–H36)	3.7	3.6	1.03 (0.93–1.14)	0.593
Glaucoma (H40–H42)	4.7	5.0	0.93 (0.85–1.02)	0.110
Disorders of vitreous body and globe (H43–H45)	0.3	0.4	0.83 (0.60–1.14)	0.254
Disorders of optic nerve and visual pathways (H46–H48)	0.2	0.2	0.93 (0.61–1.42)	0.745
Disorders of ocular muscles, binocular movement, accommodation and refraction (H49–H52)	2.4	2.3	1.02 (0.90–1.16)	0.743
Visual disturbances (H53)	9.3	9.2	1.00 (0.94–1.07)	0.932
Visual impairment including blindness (H54)	5.1	5.4	0.94 (0.86–1.03)	0.174
Having both hearing and visual impairment^1^	5.5	4.9	1.14 (1.04–1.24)	0.005
Only hearing impairment^1^	5.7	4.5	1.26 (1.15–1.38)	<0.001
Only visual impairment^1^	22.9	23.9	0.97 (0.93–1.02)	0.219
No hearing and no visual impairment	65.9	66.7	*Reference*	

*^1^Reference: patients without hearing and vision impairment. Multivariate logistic regression models were adjusted for age, sex, health insurance coverage, and the following comorbidities: diabetes mellitus, ischemic heart disease, stroke, intracranial injury, epilepsy, Parkinson’s disease, and depression*.

Vision impairment (of any type) was documented in 28.4% of patients later diagnosed with dementia compared to 28.8% of controls. Disorders of the lens (12.6% vs. 12.7%) and visual disturbances were documented most frequently (9.3% vs. 9.2%). However, vision loss was nearly equally prevalent in patients with dementia and those without dementia, without any detectable trend favoring one of these groups.

The presence of hearing impairment exclusively, without any vision impairment, was detected in 5.7% of patients with and 4.5% without dementia. By contrast, vision impairment exclusively, without any hearing impairment, was found more often in non-dementia controls (23.9%) compared to patients later diagnosed with dementia (22.9%). A combination of both hearing and vision impairment was found in 5.5% of patients with and 4.9% of patients without dementia.

### Association Between Hearing and/or Vision Loss and Dementia

Any hearing impairment (OR: 1.21, 95% CI 1.13–1.29, *p* < 0.001) as well as each separate hearing impairment diagnosis, such as conductive and sensorineural hearing loss (OR: 1.27, 95% CI 1.05–1.54, *p* = 0.015) and other hearing loss (OR: 1.20, 95% CI 1.20–1.28, *p* < 0.001) were significantly associated with an increased risk of dementia. No association was observed between any (OR = 0.98, 95% CI 0.98–1.03, *p* = 0.434) or a specific visual impairment diagnosis and dementia.

In addition, the combination of both hearing and visual impairments was significantly associated with a higher risk of dementia (OR = 1.14, 95% CI 1.04–1.24, *p* = 0.005). However, the risk of developing dementia diseases was lower compared to the group of patients who had hearing impairment exclusively (OR = 1.26, 95% CI 1.15–1.38, *p* < 0.001). Therefore, visual impairment alone was not significantly associated with dementia (OR = 0.97, 95% CI 0.97–1.02, *p* = 0.219) compared to the reference group of patients without any hearing and visual impairment. Table 2 shows the results of the multivariate regression analyses.

## Discussion

This analysis adds important evidence that contributes to the limited body of knowledge on the association between hearing and/or vision loss and dementia, demonstrating that hearing impairment only is significantly associated with a higher risk of dementia. Neither of the documented diagnoses objectively indicated that vision loss or the combination of visual and hearing impairment was significantly associated with dementia.

Several previous studies have evaluated the association between hearing loss and the risk of dementia. Loughrey et al. (2018) revealed, based on a meta-analysis of 20,264 subjects from different prospective cohort studies, that hearing impairment was significantly associated with dementia diseases (OR 1.28; CI 95% 1.02–1.59). This finding is in line with the results of the present analysis. Due to the larger sample size in our analysis, the confidence interval of our analysis was smaller and, thus, our results are more precise (Wei et al., 2017; Loughrey et al., 2018).

However, there is some evidence suggesting that vision loss is associated with an increased risk of developing dementia diseases. Based on a retrospective cohort study that included 7,685 patients, Davies-Kershaw et al. (2018) revealed that patients who rated their own vision as moderate were two times (HR 2.0, CI 95% 1.4–3.1) and those with very poor vision, which was comparable to being blind, four times (HR 4.0, CI 95% 2.6–6.1) more likely to have dementia compared to those without vision impairment. Furthermore, based on a population cohort of 7,736 initially healthy and non-dementia patients, Naël et al. (2019) revealed that moderate to severe near-vision impairment was also associated with an increased risk of dementia in the first 4 years of follow-up (HR 2.0, CI 95% 1.2–3.3), but not when patients were followed for more than 4 years. Furthermore, self-reported vision impairment was associated with an increased risk of dementia within the time frame of 4 years (HR 1.5, CI 95% 1.1–2.0), but this association was no longer significant after adjusting for important baseline covariates, such as cognitive impairment. Therefore, these results may suggest that vison loss could be associated with an increased risk in the short-term. However, results remain uncertain in the long run. Our findings showed that there was no significant association with an increased risk of dementia for any of the objective measures of vision loss. Neither refraction disorders nor severe visual impairment—including blindness—were found to be significantly associated with an increased risk of dementia diseases, which is contrary to some previous findings. The study of Davies-Kershaw et al. (2018) was based on a large nationwide sample of people aged 50 years and older. However, just 2.5% (*n* = 195) of patients in the sample were diagnosed with dementia diseases, which is well below the general population estimates for a group of the same age composition. Furthermore, the observed association was based on a much smaller sample size when compared to this analysis (Matthews et al., 2013, 2016).

Even though dementia diseases are, in fact, underdiagnosed, the low number of dementia cases in this study limits the generalizability of these findings. Furthermore, even though some studies confirmed the comparability and validity of subjective and objective measures of vision impairment (Whillans and Nazroo, 2014), the use of some listed diagnoses made by general practitioners that are related to vision loss (Davies-Kershaw et al., 2018) could be a further reason for the deviating results, especially since only a single question was used to assess the self-reported vision loss, whatever the cause of vision loss was. The information given in primary and secondary data sets could tremendously differ with respect to the identification of patients’ sensory impairments. Whereas hearing impairment is usually well-documented in general practitioners’ files, this does not apply for vision impairment, as demonstrated by this analysis. Therefore, further research is needed to evaluate if the secondary datasets are valid for the identification of sensory impairments, especially vision impairment. In addition, the ability to self-report vision loss probably varies according to cognitive capacities. This could be the reason for the inconsistent findings regarding a significant association between vision loss and the risk of dementia, which has been observed in previous studies. Therefore, further research is needed to clarify the validity of objective and subjective measures for vision loss, as well as explain the differences in their association with a higher risk of developing dementia diseases.

The results of our study demonstrated that only hearing loss is associated with a higher risk of dementia diseases and that the combination of both hearing and vision loss certainly increases sensory impairment, but not the risk of developing dementia diseases, thus demonstrating the importance of hearing ability as a protective factor against dementia patients. People with hearing loss have various neuronal changes. Hearing loss in older adults may result in an acceleration of aging because the nervous systems can alter synapses and neural anatomy (Martini et al., 2014). To compensate for the decreased auditory input that is caused by hearing loss, more listening effort is required through the additional recruitment of frontal areas (Campbell and Sharma, 2013). In hearing-impaired patients, an increase in cognitive load occurs. Consequently, cognitive processes such as memory and executive function are adversely affected (Boyle et al., 2008). Thus, hearing loss may alter the usual pattern of resource allocation in the brain, affecting neural reserves and cognitive performance (Lin et al., 2013), and leading to altered auditory processing. Hence, early onset of hearing loss in older adults may causally accelerate atrophy in the entire brain, which could lead to cognitive reserve depletion in the brain (Lin et al., 2014). The coexistence of hearing loss in patients living with dementia could cause adverse patient-related outcomes due to difficulties in participating in daily social activities and to breakdowns in communication between patients and caregivers and between patients and professionals. These challenges, in turn, can lead to social exclusion, increase stress and fatigue, and exacerbate neuropsychiatric behaviors, such as apathy, depression, and aggression (Slaughter et al., 2014; Palmer et al., 2017). All of these are related to a lower health-related quality of life.

Even though the prevalence of hearing loss in patients living with dementia is very high and the association between hearing loss and cognitive decline is well studied, the utilization of hearing aids is only moderate (Nirmalasari et al., 2017). Maharani et al. (2018) revealed that early recognition and treatment of hearing loss could have the potential to slow down cognitive decline and potentially delay the onset of dementia, and should, therefore, be a focus of healthcare providers and care management models. Therefore, hearing impairment must be identified early in older adults and in patients living with dementia, and the unmet need for effective hearing aids must be met. Collaborative care management approaches could be a potential tool for improving this situation, and thus help patients get access to such important aids, which can delay the progression of this degenerative disease. Therefore, several commissions—such as, for example, the Lancet Commission on Dementia Prevention, Intervention, and Care—highlight the importance of optimizing hearing in patients with dementia in order to improve the management of psychosis, agitation, and depression related to this condition (Livingston et al., 2017). According to the findings of previously published studies, as well as the current study, early recognition and treatment of hearing loss has the potential to delay the onset of dementia diseases and improve patients’ outcomes. It should, therefore, be a focal point for healthcare providers and care management models. However, further research is needed to evaluate the efficacy and cost-effectiveness of such hearing aids in patients where cognitive impairment or dementia and hearing loss coexist.

### Strengths and Limitations

We conducted a case-control study of patients with and without a diagnosis of a dementia disease, using data from GP practices. The diagnoses listed in the files of the general practitioners are usually used for reimbursement. Therefore, the data may not be complete, especially not for vision impairment diagnoses, which are, in most cases, not documented in general practitioners’ files. Among the elderly, the prevalence of vision impairment is approximately 70%. In this study, diagnoses related to vision impairment of any type was only documented in 28% of cases, demonstrating that vision impairment was underdiagnosed and underrepresented in this data set. This limits the generalizability of the non-significant association found between dementia and vision impairment and should, therefore, be evaluated in further research, using other objective or subjective measures.

Furthermore, hearing impairment during the cognitive assessment or dementia screening procedures might anticipate the dementia diagnoses due to the fact that patients are unable to hear and, thus, to understand questions included in the assessment correctly, even though these patients might not be cognitively impaired. This could lead to false-positive dementia diagnoses and, thus, could support the association between hearing loss and dementia. In addition, our analyses were based on the initially documented dementia diagnoses. It should be noted that the basic process for recognizing cognitive impairment is completely different compared to the process used for reimbursement purposes. Therefore, the diagnoses used in the analyses are not verified for their correctness and accuracy. This also applies to the diagnosis of hearing and vision loss. In addition, dementia diseases are underdiagnosed by general practitioners. In Germany, only 40% of PwD are formally diagnosed with dementia. This diagnosis rate of dementia in German primary care is well within the range of the international data (20%–50%; Eichler et al., 2014). This analysis was based on diagnoses listed in the files of general practitioners. Diagnoses made by specialists such as neurologists or psychiatrists, who usually handle the differential diagnosis process, were not included in this analysis, which limits the generalizability of the results.

Despite these limitations, the strength of the present study was that the analysis was based on a total of 122,708 patients treated by their general practitioners. Therefore, the sample is adequate for answering the research questions, with considerable external validity.

## Data Availability Statement

The datasets generated for this study are available on request to the corresponding author.

## Author Contributions

BM drafted the manuscript. KK is responsible for the statistical analyses. WH contributed significantly to the manuscript.

## Conflict of Interest

KK is an employee of IQVIA and has no potential conflicts of interest with respect to the research, authorship, and/or publication of this article.

The remaining authors declare that the research was conducted in the absence of any commercial or financial relationships that could be construed as a potential conflict of interest.
